# Plasmapheresis for refractory toxic epidermal necrolysis unresponsive to conventional therapy: a case report and literature review

**DOI:** 10.3389/fimmu.2025.1579349

**Published:** 2025-05-28

**Authors:** Shun-Qi Liao, Zhang-Rong Yan, Lun-Wei Lin, Ming Deng, Guo-Jin Xiao, Pei-Yang Gao

**Affiliations:** ^1^ Department of Critical Care Medicine, Hospital of Chengdu University of Traditional Chinese Medicine, Chengdu, China; ^2^ Nursing Department, Luzhou Hospital of Traditional Chinese Medicine, Luzhou, China

**Keywords:** toxic epidermal necrolysis, Steven Johnson syndrome, plasmapheresis, severe cutaneous adverse reaction, refractory

## Abstract

Toxic epidermal necrolysis (TEN) is a rare and life-threatening severe cutaneous adverse reaction. The conventional treatment approach includes immunomodulatory therapies, such as systemic corticosteroids, cyclosporine, intravenous immunoglobulin (IVIG), and tumor necrosis factor-alpha (TNF-α) inhibitors. Plasmapheresis, as a potential treatment for TEN, is rarely used in patients with refractory TEN. We report a successful case of plasmapheresis treatment in a patient with refractory TEN who did not respond to conventional treatment, and we provide a literature review. A 65-year-old female presented with diffuse erythematous papules covering her entire body, along with multiple blisters and bullae, and partial detachment of the epidermis and mucosa. The area of epidermal exfoliation exceeded 30% of the total body surface area, and Nikolsky’s sign was positive. Despite a week of methylprednisolone treatment, numerous blisters and bullae developed, and the area of epidermal exfoliation expanded to 62%. IVIG and TNF-α inhibitors were subsequently added, but the disease remained uncontrolled. Plasmapheresis treatment was initiated. Epithelial regeneration was observed after three days of plasmapheresis. After plasmapheresis was given 5 times, the patient finally recovered. This case highlights the significance of plasmapheresis in the treatment of refractory TEN, particularly when conventional therapies are ineffective. More studies are needed in the future to confirm the efficacy of plasmapheresis treatment.

## 1 Introduction

Toxic epidermal necrolysis (TEN) is a rare serious skin adverse reaction mediated by T cells (1). TEN and Stevens-Johnson syndrome (SJS) represent the same disease, differing primarily in the extent of epidermal detachment across the total body surface area (BSA): less than 10% for SJS, 10–30% for SJS-TEN overlap, and greater than 30% for TEN (2). More than 80% of TEN cases are related to drug exposure, such as anticonvulsants, antidepressants, sulfonamides, nonsteroidal anti-inflammatory drugs, anti-infectious drugs, and immune checkpoint inhibitors (3). TEN is mainly characterized by extensive skin and mucosal necrosis, often involving multiple organs or systems, and is extremely prone to cause serious infection. The mortality rate is as high as 34–50% (1, 4, 5). Experimental studies have confirmed that TEN is closely related to the apoptosis of keratinocytes caused by the immune response (6). First, the Fas/Fas ligand (FasL) complex interacts with the Fas-associated death domain protein (FADD), triggering the activation of caspases (7, 8). This process results in the disassembly of the internal structure of keratinocytes, ultimately leading to keratinocyte apoptosis (7, 8). Second, cytokines produced by activated T cells and NK cells—such as granulysin, granzyme, TNF-α, IL-2, IL-6, and IFN-γ—accumulate on susceptible skin lesions and mucosal surfaces (6, 9, 10). These cytokines exert cytotoxic effects on keratinocytes, leading to keratinocyte apoptosis. Therefore, suppressing the inflammatory response and eliminating inflammatory factors are regarded as promising therapeutic strategies for TEN.

The management strategy for TEN involves administering immunomodulating treatments following the withdrawal of the offending medication(s) and providing supportive care (11). Immunomodulatory therapies include systemic corticosteroids, cyclosporine, immunoglobulin (IVIG), and Tumor necrosis factor-alpha (TNF-α) inhibitors (12, 13). These treatments are generally effective. However, there are rare cases of refractory patients whose symptoms persist or even worsen despite immunomodulatory therapy (14). As a potential therapy for TEN, plasmapheresis has no large-scale randomized controlled trials to prove its efficacy. Research shows that plasmapheresis effectively removes pathogenic factors such as immune complexes, antibodies, and inflammatory cytokines from the plasma (15). In addition, it can also reduce the activation of T lymphocytes and B lymphocytes (15). This indicates that plasmapheresis may be a potential and valuable therapeutic approach. To date, no cases of refractory TEN treated with plasmapheresis have been reported. We present the first reported case of a patient with TEN who was refractory to conventional therapies including corticosteroids, TNF-α inhibitors, and IVIG. Plasmapheresis treatment significantly improved the condition of this patient. At the same time, this study conducted a literature review on the treatment of TEN with plasmapheresis.

## 2 Materials and methods

**Figure 1:**
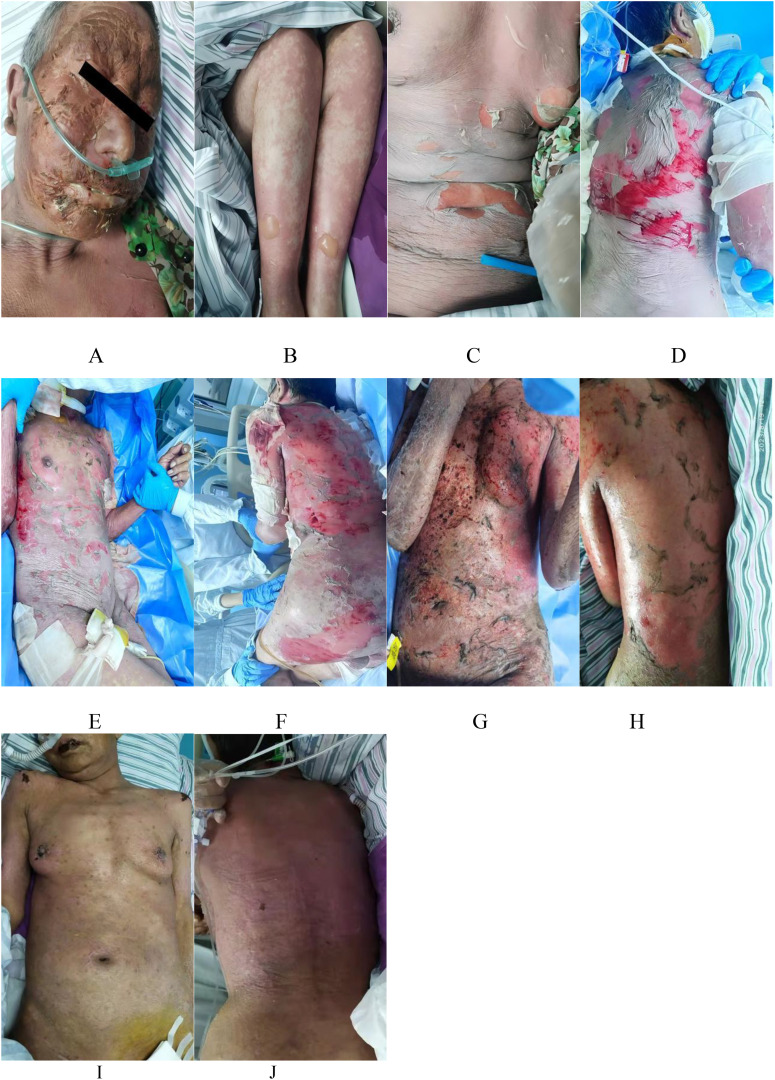
Before treatment, the skin of the patient’s face **(A)**, both lower limbs **(B)**, chest and abdomen **(C)**, and back **(D)** exhibited diffuse erythema, papules, multiple blisters, and bullae, accompanied by partial epidermal and mucosal detachment, along with a positive Nikolsky sign. Before plasmapheresis, the patient’s chest, abdomen **(E)**, back, and buttock **(F)** skin; After plasmapheresis, the patient’s chest, abdomen **(G)**, and back buttocks **(H)** skin. Discharge, The patient’s chest, abdomen **(I)**, and back **(J)** skin.

A 65-year-old female was admitted to the intensive care unit (ICU) due to diffuse erythematous papules all over her body, multiple blisters and bullae, and partial detachment of the epidermis and mucous membranes (**Figures 1A-D**). She was diagnosed with epilepsy a year before and had been undergoing long-term treatment with lamotrigine and valproate. Fifteen days before the patient’s visit, they began to experience a cough, oral mucosal ulcers, and erythema in both upper limbs accompanied by pain. She went to the local hospital for treatment and was given antitussive and analgesic treatment, but the specific therapeutic regimen was not documented. After the treatment, there was no improvement, and her condition continued to worsen, so she came to our hospital for further treatment.

Physical examination revealed widespread atypical target-like maculopapular eruptions of varying sizes across the body. The chest, abdomen, and limbs showed relaxed blisters and bullae, accompanied by areas of fused epidermal detachment and a positive Nikolsky sign. The total area of epidermal detachment accounts for 32% of the BSA. Bilateral conjunctival hyperemia was present, along with increased discharge from the corners of the eyes. Erosions of the oral mucosa were noted, with blood crusts observed on the lips. Erosions of the vulvar mucosa were accompanied by purulent discharge.

Upon admission, laboratory tests indicated the patient’s white blood cell(WBC) count of 10.04×10⁹/L, a monocyte count of 1.89×10⁹/L, a hemoglobin level of 104 g/L, a platelet(PLT) count of 312×10⁹/L, a creatinine level of 33.1μmol/L, an alanine aminotransferase (ALT) level of 100 U/L, an aspartate aminotransferase (AST) level of 107 U/L, and a C-reactive protein(CRP) level of 100 mg/L.

**Figure 2:**
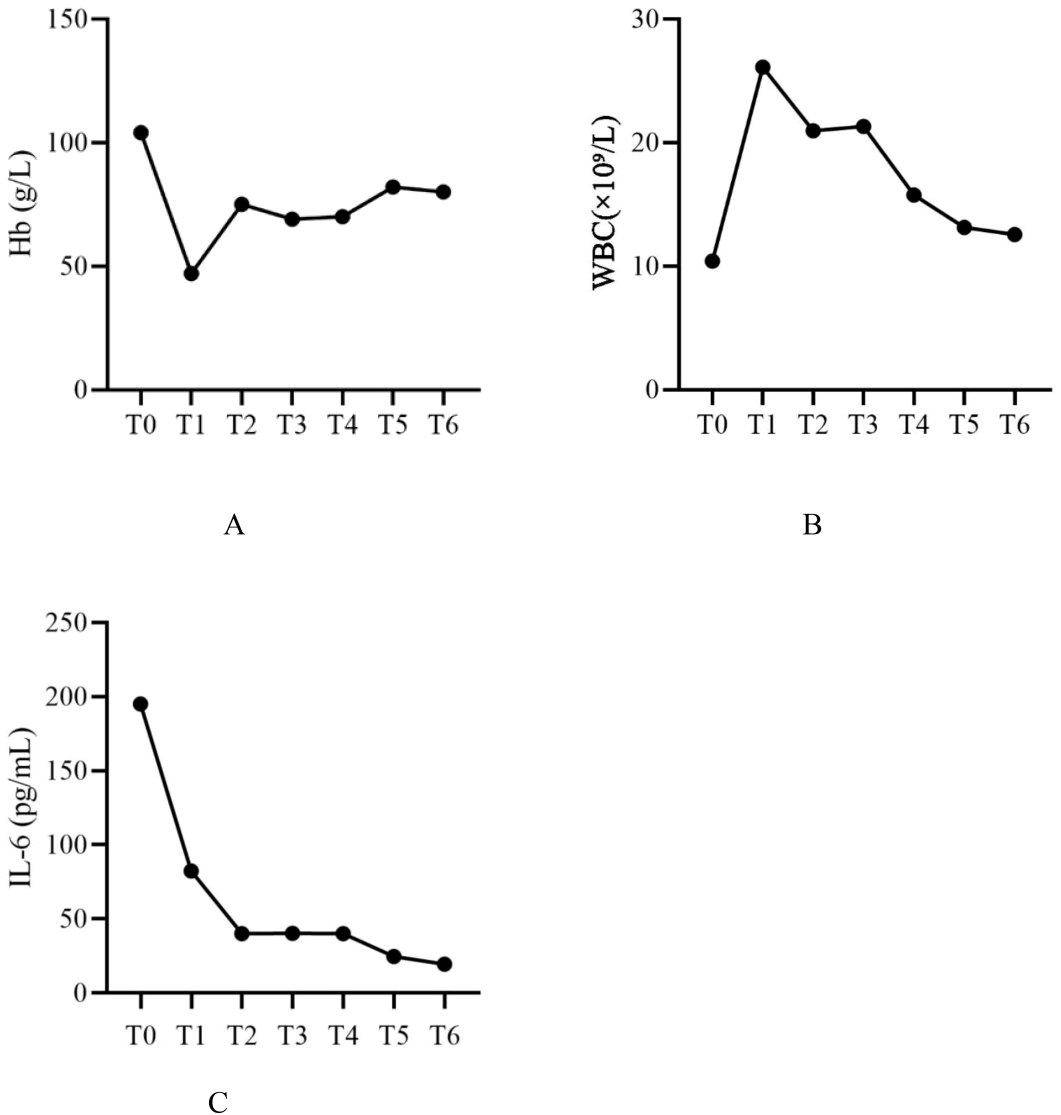
Dynamic changes of WBC, Hb, and IL-6 levels. WBC, white blood cell count; Hb, hemoglobin; IL-6, interleukin-6; T0, Before treatment; T1, Before plasmapheresis treatment; T2, After the 1st plasmapheresis treatment; T3, After the 2st plasmapheresis treatment; T4, After the 3st plasmapheresis treatment; T5, After the 4st plasmapheresis treatment; T6, After the 5st plasmapheresis treatment.

During hospitalization, the patient’s hemoglobin levels showed a significant decline, reaching a minimum of 47 g/L. Subsequently, with ongoing treatment, levels gradually increased, rising to 80 g/L after the final plasmapheresis treatment (**Figure 2A**). The WBC count peaked at 26.1 ×10⁹/L and decreased to 12.56 ×10⁹/L after the final plasmapheresis (**Figure 2B**). IL-6 was 195.16 pg/mL upon admission. During plasmapheresis treatment, IL-6 gradually decreased, reaching 19.51 pg/mL after the last plasmapheresis treatment (**Figure 2C**). The dynamic changes of various laboratory parameters are shown in **Table 1**.

**Table 1:** Longitudinal changes in laboratory parameters during plasmapheresis treatment.

Parameter	Before treatment	Before plasmapheres treatment	After the 1st plasmapheresis treatment	After the 2st plasmapheresis treatment	After the 3st plasmapheresis treatment	After the 4st plasmapheresis treatment	After the 5st plasmapheresis treatment
IL-6 (pg/mL)	195.16	82.4	40.06	40.22	40.06	24.66	19.51
CRP (mg/L)	100	187.9	150.05	97.7	39.43	13.84	10.69
Cr (μmol/L)	33.1	25.7	28.8	28.6	27.9	23.1	28.34
UREA (mmol/L)	4.15	6	7.3	4.1	3.85	3.88	4.95
ALB (g/L)	27.6	25.3	46.5	25.7	27.7	30.1	30.7
ALT (U/L)	100	70	59	47	66	51	47
AST (U/L)	107	31	30	31	32	24	21
TBIL (μmol/L)	18.1	16.8	9.4	13.9	10.3	7.6	8.7
PT (s)	15.3	15.4	13.7	13.9	13.7	13.2	12.8
APTT (s)	34.6	37.5	41.7	43	33.7	32.5	34.5
WBC(×10⁹/L)	10.4	26.1	20.97	21.32	15.76	13.13	12.56
MONO (×10⁹/L)	1.89	0.96	0.2	0.54	0.49	0.53	0.66
RBC (×1012/L)	3.35	1.55	2.48	2.24	2.37	2.69	2.6
Hb (g/L)	104	47	75	69	70	82	80
PLT (×10⁹/L)	312	321	205	136	133	140	142

IL-6, Interleukin-6; CRP, C-reactive protein; Cr, creatinine; UREA, urea; ALB, albumin; ALT, alanine aminotransferase; AST, aspartate aminotransferase; TBIL, total bilirubin; PT, prothrom; APTT, activated partial thromboplastin time; WBC, white blood cell count; MONO, monocyte count; RBC, red blood cell count; Hb, hemoglobin; PLT, platelet count.

The pathogenic examination indicated no abnormalities for herpes simplex virus, varicella-zoster virus, Epstein-Barr virus, cytomegalovirus, and rubella virus. A skin biopsy could not be performed due to family refusal. However, based on the patient’s medical history, clinical presentation, laboratory findings, atypical skin lesions, and positive Nikolsky sign, the diagnosis was strongly considered to be TEN. Complications included liver dysfunction and hypoproteinemia. The Score of Toxic Epidermal Necrolysis (SCORTEN) was 3, indicating a predicted mortality rate of 12%.

**Figure 3:**
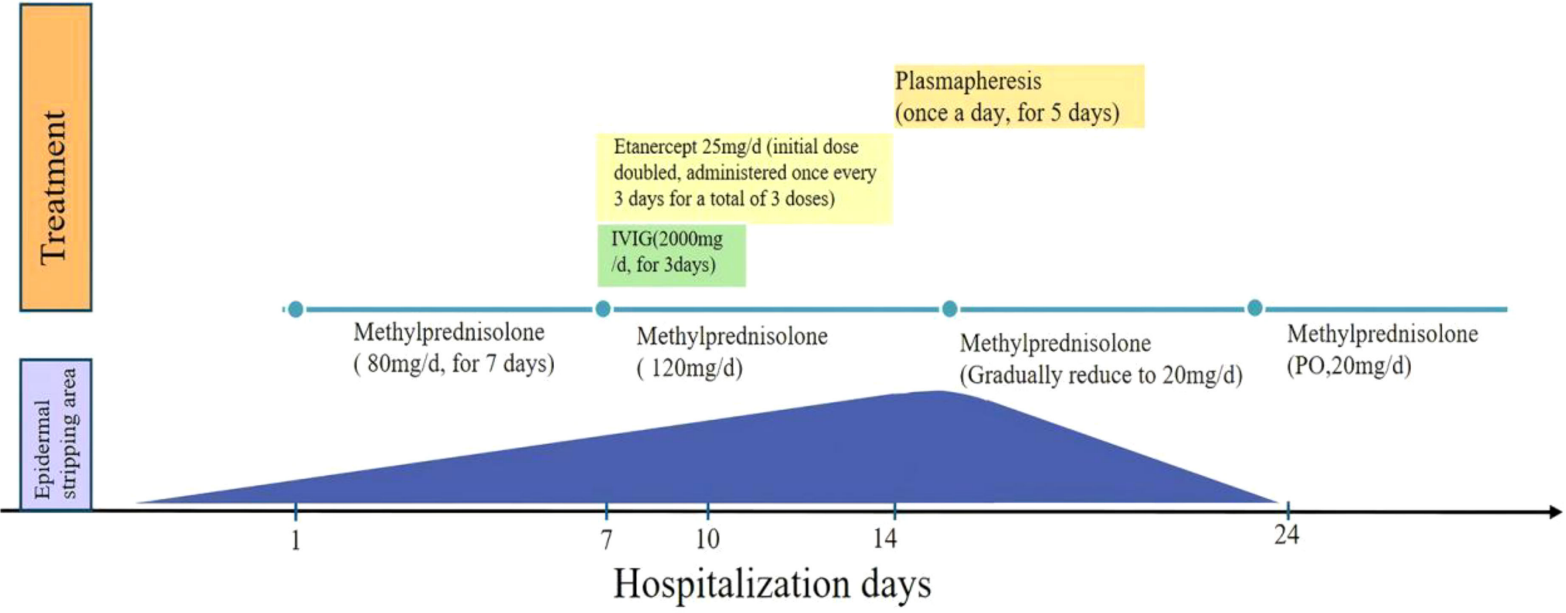
Medication time point diagram. The blue area indicates the degree of epidermal detachment during hospitalization. IVIG, intravenous immunoglobulin.

Due to the patient’s poor baseline condition and rapid disease progression, intravenous methylprednisolone at 80 mg/day was administered for 7 days. After 7 days of treatment, the skin lesions were not controlled, and many blisters and bullae appeared, with epidermal detachment accounting for 62% of BSA. Additionally, there was a complication of sepsis. On the eighth day of treatment, the patient was administered methylprednisolone at 120 mg/day, immunoglobulin at 2 g/day (once daily for three days), and the TNF-α inhibitor (Etanercept) at 25 mg/day (initial dose doubled, administered once every three days for a total of three doses) (**Figures 1E, F**). However, the disease was still not under control. Plasmapheresis (once a day, 5 times in total) was started on the 14th day. The volume of plasmapheresis was 2L each time. Most of the skin started epithelialization 3 days after plasmapheresis (**Figures 1G, H**). Following the improvement in the patient’s condition, the methylprednisolone dosage was gradually tapered to 20 mg/day. The patient was discharged after 24 days of treatment, and methylprednisolone tablets 20mg/d were taken orally (**Figures 1I, J**). **Figure 3** shows the trend of our treatment plan and skin involvement area.

## 3 Literature review

### 3.1 Search strategy

We searched PubMed, Web of Science, Wanfang, and CNKI databases for case reports of plasmapheresis-assisted treatment of SJS/TEN published from inception to November 24, 2024. The search terms used were combined text, and the above databases were retrieved using the keyword + free word search strategy: (Stevens-Johnson syndrome/toxic epidermal necrolysis/SJS/TEN) and (Plasmapheresis/Plasma Exchange/Blood Component Removal/Extracorporeal Circulation). We used equivalent translations of the same search term to retrieve case reports from the Chinese database. We only considered case reports and excluded original studies, surveys, conference abstracts, editorials, and reviews.

### 3.2 Inclusion and exclusion criteria

1) SJS/TEN diagnosed according to clinical and/or histopathological criteria (16); 2) The description of plasmapheresis during treatment is clear.

### 3.3 Data collection

**Figure 4:**
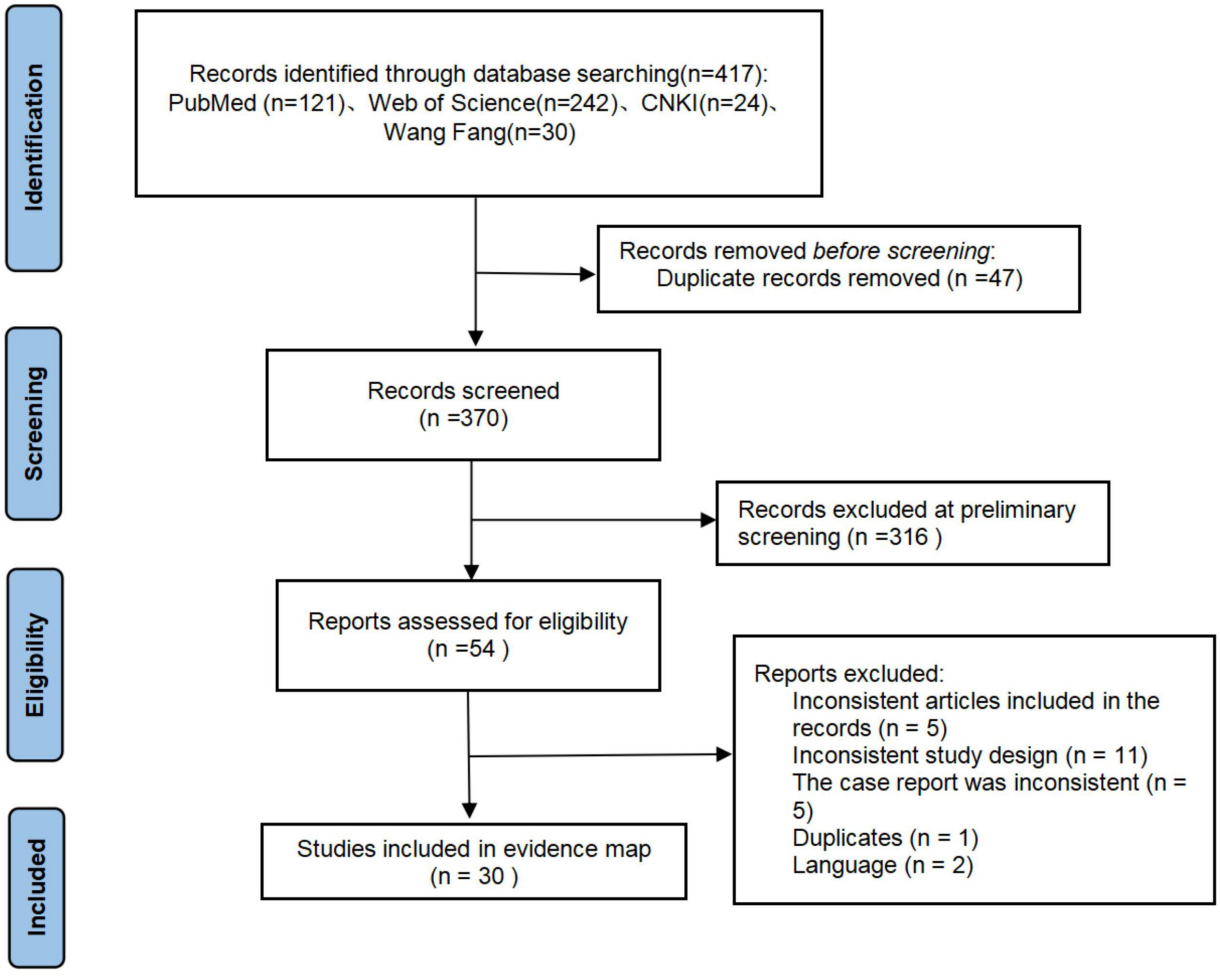
PRISMA flow diagram detailing the selection process.

Two researchers independently screened the literature and extracted the data separately. If a conflict occurs, it is resolved by reaching a consensus or by a third researcher. The PRISMA flowchart summarizes the literature search and final research choices (**Figure 4**). The following information is extracted from the report using a predefined data extraction template. Patient information included gender, age, country, initial disease, predisposing factors, severe cutaneous adverse reaction (SCAR) type, SCORTEN score, time to admission, length of stay (LOS), description of plasmapheresis, author’s views, and outcome.

### 3.4 Results

#### 3.4.1 Study selection

A total of 417 articles were retrieved from the literature search, 47 duplicate articles were deleted, 316 articles were deleted after reading the full text, 5 articles were not in line with the article, 11 articles were not in line with the study design, 5 articles were not in line with the case report requirements, 1 article was repeated, and 2 articles were not in English. Finally, 30 case reports were obtained from 56 patients from 10 countries (17–46).

#### 3.4.2 Clinical characteristics

In this study, combined with our current cases, a total of 57 patients were included. Among the 57 patients, 48 (84.2%) had TEN, 6 (10.5%) had SJS and 3 (5.3%) had SJS-TEN overlap. Among all the included cases, about 47.4% (n=27) were female and 52.6% (n=30) were male. The age ranged from 1 to 78 years old, with a median age of 28 years old. A total of 52 patients (91.3%) survived. 41 cases (71.9%) were cured, 9 cases (15.8%) improved, and 5 cases (8.8%) died. Among the 36 cases reporting underlying conditions, 17 cases (47.2%) had co-infections. Seven cases (19.4%) were diagnosed with cancer. Two cases (5.5%) were infected with HIV. Two cases (5.5%) were diagnosed with epilepsy and were undergoing antiepileptic treatment. Two cases (5.5%) were diagnosed with tuberculosis. A total of 32 case reports identified the triggering drugs, including antibiotics in 10 cases (31.2%), antiepileptic drugs in 6 cases (18.7%), immune checkpoint inhibitors in 5 cases (15.6%), and nonsteroidal anti-inflammatory drugs in 4 cases (12.5%). The average LOS of patients was 19.57 days, and the number of individual plasmapheresis treatments ranged from 1 to 13 times, with an average of 4.5 times. Among the 30 case reports included, 21 authors believed that plasmapheresis was beneficial, and 9 authors believed that plasmapheresis might be beneficial. Among the 15 TEN patients with clearly documented timing of plasmapheresis, 12 patients initiated plasmapheresis early (within 72 hours of admission), while 5 patients initiated plasmapheresis late (>72 hours). Among the cases with clearly documented prognosis, 10 patients who initiated plasmapheresis early were cured, and 2 patients showed improvement. Both patients who initiated plasmapheresis late were cured. The average length of hospital stay was 21.27 days for patients who initiated plasmapheresis early and 33 days for those who initiated plasmapheresis late. For more detailed information on the overall demographic and clinical characteristics of patients, please refer to **Supplementary Table 1**.

## 4 Discussion

TEN is considered a life-threatening medical emergency, and early recognition and appropriate management are essential for survival. Systemic corticosteroid therapy is regarded as the first-line treatment with anti-inflammatory and immunosuppressive effects (47). However, evidence indicates that corticosteroids can enhance protein catabolism, delay epithelialization, and increase susceptibility to sepsis (48). Sepsis, a common complication of TEN, has been identified as the leading cause of mortality in TEN (49). Therefore, the prevention and treatment of sepsis is very important. The patient in this case developed sepsis during methylprednisolone treatment, likely due to excessive immunosuppression, which temporarily masked the early symptoms of sepsis (50). At the same time, patients who continue to receive corticosteroids may develop hormone resistance (51). To manage unresponsiveness to corticosteroid therapy, various immunosuppressants are used in combination with corticosteroids as an alternative (14). The treatment plan for this case was adjusted promptly, with the addition of TNF-α inhibitor and IVIG. However, the patient’s skin detachment continued to worsen. TNF-α inhibitor exerts anti-inflammatory effects by inhibiting TNF-α (52). IVIG may play a role by inhibiting Fas receptors (52). However, the destruction of epidermal cells is caused by several apoptotic pathways and the blockade of Fas-FasL interaction is insufficient. In addition, the optimal timing for immunosuppressive therapy remains unclear, which may significantly diminish its efficacy.

Considering the worsening progression despite ongoing treatment, plasmapheresis treatment was introduced. After using plasmapheresis, both systemic and skin conditions began to improve and effectively stabilized the circulation and respiration. The patient was subsequently discharged from the ICU. Plasmapheresis is an *in vitro* blood purification technology, which uses a blood cell separator to separate plasma from blood cells. It can not only remove harmful molecules but also supplement protective plasma proteins(See **Supplementary Figure 1** for the mechanism of plasmapheresis) (53). The case reported in this study showed a limited response to corticosteroids, TNF-α inhibitors, and IVIG. Additionally, the onset of sepsis led to the continued worsening of the disease. Plasmapheresis quickly removes FasL, various autoantibodies, antigen-antibody complexes, toxic metabolites of medications, and inflammatory mediators (such as complement, C-reactive protein, IL-2, IL-6, TNF-α, interferon-γ) from the plasma, effectively reversing the course of the disease (15). It is particularly beneficial in the treatment of sepsis and TEN. A randomized controlled trial confirmed that plasmapheresis treatment can effectively stabilize hemodynamics and improve lactate clearance rates (54). In this case, following plasma exchange therapy, the patient experienced significant improvement in systemic inflammatory response (with IL-6 levels decreasing from 82.4 pg/mL to 19.51 pg/mL) and skin lesions. Circulatory and respiratory functions also showed signs of stabilization. In addition, lowering plasma pro-inflammatory cytokines can also control T lymphocyte activation and the inflammatory response, which is crucial for the immune system’s recuperation and the prognosis of patients (15). Equally important, plasmapheresis replenishes fresh frozen plasma to replenish coagulation factors, fibrinolysin, angiopoietin, immunoglobulin, and so on (54). This process effectively promotes wound healing, enhances immunity, and maintains fluid balance.

Studies reported that plasmapheresis treatment can effectively save patients with TEN who have developed resistance to corticosteroid treatment, and the disease did not rebound after using it (55, 56). A case of TEN induced by immune checkpoint inhibitors showed poor outcomes after one month of conventional treatment, complicated by sepsis. The patient recovered fully after five sessions of plasmapheresis therapy (17). This suggests that plasmapheresis can play a crucial role, potentially life-saving, in refractory TEN patients, especially those with concurrent sepsis. Another observational study found no significant benefit in terms of mortality or length of hospital stay after plasmapheresis treatment (52). However, this study had several confounding factors, such as an unclear distinction between refractory patients and the stage of plasmapheresis treatment (57).

During plasmapheresis, the patient’s platelet count exhibited an initial decline followed by stabilization. This dynamic profile may be attributed to multiple mechanisms. The extracorporeal circuit mechanically consumed platelets, while high-dose heparin (4 mL IV bolus followed by 4 mL/h maintenance) directly inhibited platelet function (53). As a result, the platelet count significantly decreased after the first two plasmapheresis treatments. Sepsis-associated inflammatory cytokines further exacerbated platelet consumption by activating endothelial cells and coagulation cascades, promoting microthrombus formation (58). The heparin dose was reduced from the third session onward (2 mL IV bolus followed by 2 mL/h maintenance). At the same time, inflammatory mediators were cleared through plasmapheresis. These interventions alleviated platelet suppression and facilitated gradual platelet count recovery. These findings underscore the synergistic benefits of anticoagulation protocol optimization and inflammation control in restoring coagulation homeostasis.

During the hospitalization of the patient in this case, the hemoglobin level significantly decreased from 104 g/L to 47 g/L. This severe decline was due to several factors. Extensive skin and mucosal damage led to chronic blood and protein loss. Systemic inflammatory response caused erythropoiesis inhibition and inflammatory hemolysis (59). Additionally, abnormal liver function and hypoproteinemia further impaired iron metabolism and hemoglobin synthesis (60). To treat the severe anemia, the patient received a red blood cell transfusion to improve oxygen delivery. After treatment, the hemoglobin level increased to 91 g/L.

Through a comprehensive review of the literature, we compiled previously published case reports on the use of plasmapheresis for the treatment of SJS/TEN (**Supplementary Table 1**). A total of 57 patients were included, with 84.2% diagnosed with TEN (48 cases), 10.5% with SJS (6 cases), and 5.3% with SJS-TEN overlap (3 cases), making TEN the most common condition. The average LOS was 19.57 days, with an average of 4.5 plasmapheresis sessions. The overall survival rate was 91.3%, which is consistent with findings from previous studies, suggesting that plasmapheresis treatment may improve prognosis (57). The literature review indicates that 80% of TEN patients underwent plasmapheresis within 72 hours of admission, achieving a recovery rate of 83.3% (10/12) with no deaths reported. In contrast, although all patients who started plasmapheresis later survived, their average LOS was extended to 33 days. Early plasmapheresis treatment may reduce the risk of secondary infections and multiple organ failure by rapidly blocking the “cytokine storm” and reducing the toxicity of corticosteroids (61). Similarly, in diseases such as myasthenia gravis and Hodgkin’s lymphoma, early plasmapheresis has been proven to significantly improve prognosis (62, 63). This further supports the central role of the “time window” in immunomodulatory therapy. However, the optimal application time of plasmapheresis treatment still needs to be comprehensively judged according to the specific condition of patients, such as immune status and inflammatory factor levels. More clinical studies are still needed in the future to clarify the optimal interventional window of plasmapheresis treatment.

Further literature analysis revealed that among patients with underlying diseases, 47.2% had concurrent infections, 19.4% suffered from cancer, and 5.5% were HIV-infected. Infections may activate innate immunity through pathogen-associated molecular patterns (PAMPs), exacerbating T cell-mediated keratinocyte apoptosis (9). Cancer patients, due to immunosuppressive therapy or immune checkpoint inhibitors experience excessive T cell activation, increasing the risk of TEN (12). Among drug triggers, antibiotics (31.2%), antiepileptic drugs (18.7%), immune checkpoint inhibitors (15.6%), and non-steroidal anti-inflammatory drugs (12.5%) were the main driving factors. The mechanisms involve the abnormal presentation of drug-host protein complexes and the release of cytotoxic granules (6).

This study has some limitations. Plasmapheresis treatment was initiated after corticosteroids, TNF-α inhibitors, and IVIG treatment had no significant therapeutic effect. It is difficult to determine the best time to start treatment with plasmapheresis. At the same time, it is difficult to accurately evaluate the exact contribution of plasmapheresis to treatment.

Refractory TEN is a rare but growing challenge. This case report provides evidence for the use of plasmapheresis for the treatment of refractory TEN. Plasmapheresis may represent an effective and safe treatment for patients with refractory TEN. More randomized controlled trials are needed to further clarify the efficacy of plasmapheresis in the treatment of TEN.

## Data Availability

The original contributions presented in the study are included in the article/**Supplementary Material**. Further inquiries can be directed to the corresponding author.
