# Supplementary material for: Plasmapheresis for refractory toxic epidermal necrolysis unresponsive to conventional therapy: a case report and literature review
Source: Front Immunol. 2025 May 28;16:1579349. doi: 10.3389/fimmu.2025.1579349 (PMC12152979; doi:10.3389/fimmu.2025.1579349)
Supplement: Supplementary file 1 [file DataSheet1.pdf]

Supplementary Material

1 Supplementary Figures

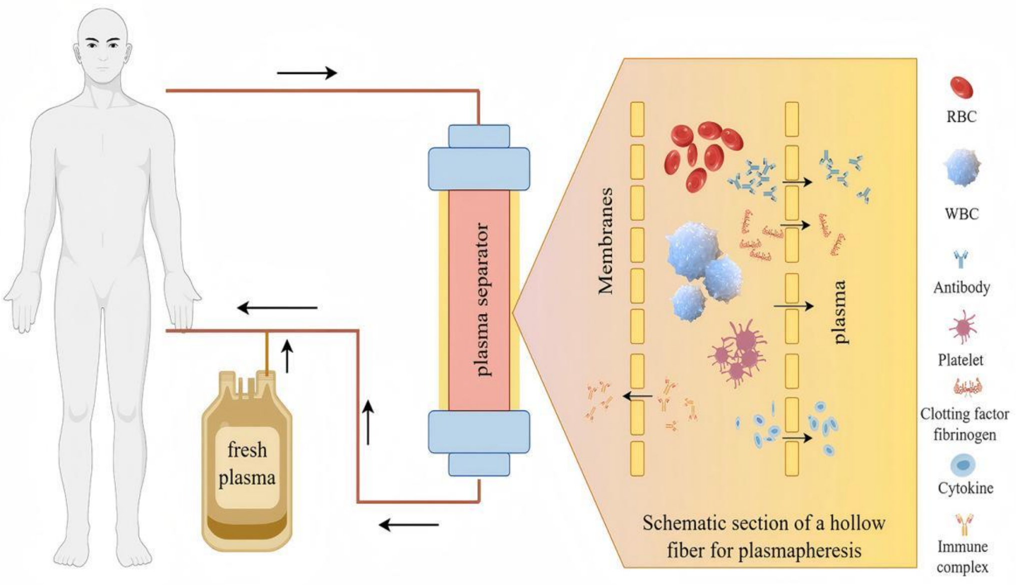

Supplementary Figures 1. Mechanism of plasmapheresis

2 Supplementary Tables

Supplementary Table 1. The clinical features of plasmapheresis in the treatment of Stevens-Johnson syndrome (SJS) and toxic epidermal necrolysis (TEN) were previously reported.

Author and year	Cases	Sex	Age(years)	State	Original disease	Precipitating factor	SCARs type	SCORTEN score	Time to admission	LOS	Description of plasmapheresis	Treatment evaluation	Outcome
Cong HY 2024(1)	2	M; F	68;61	China	Mediastinal small cell carcinoma; Central squamous cell carcinoma of the left lung	Immune checkpoint inhibitors	SJS progressed to TEN; TEN	3;5	4d;15d	19d;25d	Four times on the 3rd,4th,6th, and 13th day; Three times on the 3rd,4th, and 5th day	Beneficial	Cured; Cured
Chen XX 2024(2)	1	M	78	China	Esophageal cancer	Immune checkpoint inhibitors	TEN	NA	5d	16d	Plasmapheresis on the 2nd and 5th days, a total of twice	Beneficial	Cured
You-Wen Tan 2024(3)	1	F	35	China	COVID-19	Acetaminophen	SJS/TEN	NA	7d	60d	DPMAS, Use once every 48 hours, a total of 7 times	Beneficial	Cured
Xiaoqian Chen 2024(4)	3	M; M; F	6;9;7	China	NA	Aminophenazone; Lysine Acetylsalicylate; Ibuprofen; Chinese medicine	TEN;TEN;TEN	3;3;4	3d;2d;2d	48d;24d;79d	5 times,50–80 mL/kg;50–80 mL/kg for 5 days; 50–80 mL/kg for 5 days	Probably beneficial	Death; improvement; improvement
Han Ting 2023(5)	1	F	6	China	NA	COVID-19 inactivated vaccine	TEN	NA	3h	40d	Plasmapheresis on the 3rd and 4th day, 1000 mL/time	Probably beneficial	Cured

Zhu Qing 2022(6)	1	M	20	China	HIV	Efavirenz	TEN	NA	4d	38d	Plasmapheresis on the 19th day, 2000 mL/time	Beneficial	Cured
Sujana Reddy 2022(7)	1	F	41	USA	Alcoholic cirrhosis portal hypertension; Gastroesophageal reflux disease	Torsemide	TEN	NA	4d	Unclear	3L Plasmapheresis	Probably beneficial	Death
Yousef S Abuzneid 2022(8)	1	F	23	Pakistan	Major depressive	Carbamazepine	TEN	4	NA	28d	6 times	Probably beneficial	Cured
Wahyu Lestari 2022(9)	1	F	40	Indonesia	Type 2 diabetes mellitus; Tuberculosis	Antituberculosis therapy	TEN	2	7d	8d	Two cycle plasmaphereses,1.2L/ time	Beneficial	Improvement
Yun Cui 2022(10)	3	M; M; M	3;1;1	China	Hyperpyrexia; Severe pneumonia; Community-acquired pneumonia	Ceftriaxone; Amoxicillin-clavulanic acid	TEN; TEN; TEN	NA	0d;0d;0d	33d;18d;26d	The range number of TPE sessions was 3-5 and the duration of CHF was from 120 h to 202 h.	Beneficial	Cured; Death; Cured
Fatih Varol 2022(11)	2	F; M	6;6	Turkey	COVID-19; COVID-19	NA	TEN; SJS	3;2	3d；3d	21d;12d	TPE for 3 times (50 mL/min); TPE on the 7 th day ,3 times	Beneficial	Cured; Improvement
Oktay Perk 2021(12)	1	M	20 months	Turkey	Langerhans Cell Histiocytosis (Letterer-Siwe)	Toxic-dose vinblastine	SJS/TEN	NA	NA	28d	3 times	Beneficial	Cured
Krajewski A 2021(13)	1	F	76	Poland	COVID-19	Metamizole	TEN	NA	<1d	14d	5 cycles of TPE	Probably beneficial	Cured
Ming-Zhu Gui 2021(14)	1	F	9	China	Fever	Ibuprofen	SJS	NA	5d	26d	2 times (1,400mL/time)	Beneficial	Cured

Jiashun Cao 2021(15)	1	M	69	China	Esophagogastric junction carcinoma	Pembrolizumab	SJS/TEN	NA	NA	39d	Plasmapheresis (2,000 mL)	probably beneficial	Cured
Andrzej Krajewski 2020(16)	1	F	65	Poland	mellitus type I, hypertension, and psoriasis	Acetaminophen	TEN	NA	NA	NA	once on the 1st day, twice a day on the 2nd / 3rd day, and once on the 4th day, A total of 6 times of TPE	Probably beneficial	Cured
Hiroyo HASHIMOTO 2019(17)	1	F	40	Japan	Infertility	Cabergoline, medroxyprogesterone acetate, clomiphene, and intravenous human chorionic gonadotropin	SJS	NA	4d	41d	5 times	Beneficial	Improvement
Lu Li 2019(18)	1	M	6	China	Severe jaundice, hepatomegaly, and an annular old vesiculobullous rash	Amoxicillin and naproxen	SJS	NA	Unclearly	90d	The specific number of times is unknown	Probably beneficial	Cured
Cao Li 2019(19)	1	M	41	China	Upper respiratory tract infection	Ciprofloxacin	TEN	NA	3d	30d	The first round of TPE was on days 1, 2, 4, 5, and 6;The second round of TPE was on the 20th, 22nd, 23rd, and 24th day.	Beneficial	Cured
Rao SJ 2019(20)	2	M;F	61;55	China	Nephrolithiasis; Interstitial pneumonia, renal insufficiency	NA	TEN	NA	15d；NA	14d；24d	3 times	Beneficial	Cured; Cured
Han Feng 2018(21)	17	9M;8F	36.1 ± 25.4	China	NA	NA	TEN	2.1 ± 1.24	NA	12.1 ± 5.7(d)	Pure one-time plasmapheresis (60 mL/kg)	Beneficial	15 cases were cured, 1 case was effective

													and 1 case died.
Santosa 2018(22)	1	M	58	Indonesia	Toothache	Methampyrone	TEN	3	Unclearly	8d	TPE on the third day, once every two days,3 times	Beneficial	Improvement
Benjamin Jarrett 2016(23)	1	M	60	USA	Breast cancer and hepatitis C-induced liver cirrhosis	Docetaxel and cyclophosphamide	SJS	NA	21d	15d	Three cycles of plasmapheresis	Probably beneficial	Death
Po-Cheng Hung 2014(24)	1	F	4	China	Epilepsy	Phenobarbital	SJS	NA	NA	NA	Plasmapheresis on the 13th day,3 times, 1.5 L / time	Beneficial	Cured
Yukoh AIHARA 2012(25)	1	M	4	Japan	West syndrome	Valproate, phenytoin, clonaze-pam, risperidone, zonisamide and gabapentin<br><br>phenytoin	TEN	NA	NA	NA	6 times	Beneficial	Cured
Milan Kostal 2012(26)	4	M;M;F;F	17;58;25;18	Czech	Ureterolithiasis and relapsing urinary infection; Dysuria secondary to prostatic hypertrophy; Vulvovaginitis	Roxithromycin; Sulfamethoxazole and trimethoprim; Fungicidin; Amoxicillin and clavulanic acid	TEN	NA	3d;2d;6d;3d	24d;24d;16d;15d	The 12th to 22 days, once every other day, 6 times; the first day, once every other day,8 times; the second day, once every other day, 4 times; the second day, once a day,3 times	Beneficial	Improvement, Cured, Cured, Cured
Wojciech Szczeklik 2010(27)	1	F	31	Poland	Psoriasis	Sulphasalzine, clarithromycin, and omeprazole	TEN	NA	NA	21d	Plasmapheresis immediately, once a day (4-5 units of fresh frozen plasma) for 8 days	Beneficial	Cured
Johan Arvidson 2007(28)	1	M	9.5	Sweden	Acute lymphoblastic leukemia	Allogeneic stem-cell transplantation	TEN	NA	17d	20d	Plasmapheresis once a day on the 1st, 2nd, and 3rd days; After the deterioration of the disease, plasmapheresis was	Probably beneficial	Improvement

										performed once a day for a total of 10 times.		
Toshifumi Nomura 2004(29)	1	M	41	Zambia	Acquired Immunodeficiency Syndrome urinary and miliary tuberculosis	Isoniazid, rifabutin, ethambutol hydrochloride, and pyrazinamide trimethoprim–sulphamethoxazole	TEN	NA	NA	NA	Plasmapheresis for three consecutive days	Beneficial	Cured
Hiromichi Yamada 1998(30)	1	F	41	Japan	Exsudativum erythema	Sedes-G (isopropylantipyrin, arylisopropylacetoureid, and phenacetinum)	TEN	NA	0d	42d	DFPP on the 9th, 10th and 15th day, 3 times;2000ml / time	Beneficial	Cured

Note: SJS, Stevens-Johnson syndrome; TEN, toxic epidermal necrolysis; SCAR, severe cutaneous adverse reaction; SCORTEN, score of toxic epidermal necrolysis; LOS, length of stay; M, man; F, female; NA, Not Available; DPMAS, double plasma molecular adsorption system; HIV, human immunodeficiency virus; TPE, therapeutic plasma exchange; CHF, continuous hemofiltration; DFPP, Double Filtration Plasmapheresis

1. Cong HY, Cui HW, YU TW, Chen XG, Li J, Dong X. Therapeutic Plasma Exchange for Immune Checkpoint Inhibitor-Associated Toxic Epidermal Necrolysis: Two Case Reports *Chin J Cancer Biother* (2024) 31(02):201-6. doi: 10.3872/j.issn.1007-385x.2024.02.013.
2. Chen XX, Qin J. A Case Report of Toxic Epidermal Necrolysis Caused by Sintilimab. *Journal of Modern Medicine & Health* (2024) 40(08):1431-5.
3. Tan YW, Liu LP, Zhang K. Double Plasma Molecular Adsorption System for Stevens-Johnson Syndrome/Toxic Epidermal Necrolysis: A Case Report. *World J Clin Cases* (2024) 12(7):1371-7. Epub 2024/03/25. doi: 10.12998/wjcc.v12.i7.1371.
4. Chen X, Jiang S. Toxic Epidermal Necrolysis Complicated with Respiratory Failure in Children: A Case Report. *Heliyon* (2024) 10(4):e25830. Epub 2024/02/21. doi: 10.1016/j.heliyon.2024.e25830.
5. Han T, Sun XQ. Toxic Epidermal Necrolysis in a Child after Covid-19 Vaccination:A Case Report. *China journal of Leprosy and Skin Diseases* (2023) 39(03):195-7. doi: 10.12144/zgmfskin202303195.

6. Zhu Q, Zheng F. Hiv Infection Complicated with Toxic Epidermal Necrolysis: A Case Report. *Chinese Journal of Difficult and Complicated Cases* (2022) 21(05):533-4. doi: 10.3969/j.issn.1671-6450.2022.05.019.
7. Reddy S, Aron BK, Stewart J. A Life-Threatening Case of Torsemide-Induced Toxic Epidermal Necrolysis Associated with the Treatment of Anasarca. *Cureus* (2022) 14(3):e22895. Epub 2022/04/12. doi: 10.7759/cureus.22895.
8. Abuzneid YS, Alzeerelhouseini HIA, Rabi D, Hilail I, Rjoob H, Rabee A, et al. Carbamazepine Induced Stevens-Johnson Syndrome That Developed into Toxic Epidermal Necrolysis: Review of the Literature. *Case Rep Dermatol Med* (2022) 2022:6128688. Epub 2022/05/17. doi: 10.1155/2022/6128688.
9. Lestari W, Vella V, Yasir T, Zulfikar T. Case Report: A Successful Case of Toxic Epidermal Necrolysis Treated with Plasmapheresis Therapy. *F1000Res* (2022) 11:995. Epub 2023/05/02. doi: 10.12688/f1000research.125050.1.
10. Cui Y, Shi J, Wang C, Zhou Y, Wang F, Miao H, et al. Sequential Blood Purification for Pediatric Fatal Toxic Epidermal Necrolysis: A Case Series. *Blood Purif* (2022) 51(7):600-7. Epub 2021/09/07. doi: 10.1159/000517088.
11. Varol F, Can YY, Sahin E, Durak C, Kilic A, Sahin C, et al. The Role of Treatment with Plasma Exchange Therapy in Two Pediatric Toxic Epidermal Necrolysis Cases Related to Covid-19. *J Clin Apher* (2022) 37(5):516-21. Epub 2022/07/07. doi: 10.1002/jca.21997.
12. Perk O, Kendirli T, Dinçaslan H, Azapağası E, Meral G. Overlap Stevens Johnson Syndrome/Toxic Epidermal Necrolysis Developed Due to the Use of Toxic-Dose Vinblastine in Case of Langerhans Cell Histiocytosis(Letterer-Siwe). *Transfus Apher Sci* (2021) 60(5):103173. Epub 2021/07/04. doi: 10.1016/j.transci.2021.103173.
13. Krajewski A, Mlynska-Krajewska E, Kaczynska K, Strużyna J, Mazurek MJ. Covid-19 and Ten Treated with Ivig and Total Plasma Exchange: Simultaneous Systemic Treatment for Both Diseases. *J Investig Allergol Clin Immunol* (2021) 31(6):522-3. Epub 2021/04/14. doi: 10.18176/jiaci.0692.
14. Gui MZ, Ni M, Yin XD, Zhang T, Li ZL. Ibuprofen Induced Stevens-Johnson Syndrome and Liver Injury in Children: A Case Report. *Transl Pediatr* (2021) 10(6):1737-42. Epub 2021/07/24. doi: 10.21037/tp-21-8.
15. Cao J, Li Q, Zhi X, Yang F, Zhu W, Zhou T, et al. Pembrolizumab-Induced Autoimmune Stevens-Johnson Syndrome/Toxic Epidermal Necrolysis with Myositis and Myocarditis in a Patient with Esophagogastric Junction Carcinoma: A Case Report. *Transl Cancer Res* (2021) 10(8):3870-6. Epub 2022/02/05. doi: 10.21037/tcr-21-470.
16. Krajewski A, Mazurek MJ, Młyńska-Krajewska E. Successful Therapy of Recurrent Toxic Epidermal Necrolysis Using Total Plasma Exchange, Continuous Venovenous Hemodiafiltration, and Intravenous Immunoglobulin-Case Report. *Dermatol Ther* (2020) 33(4):e13442. Epub 2020/04/21. doi: 10.1111/dth.13442.
17. Hashimoto H, Miyachi H, Kataoka K, Maru Y, Togawa Y, Matsue H. Case of Fertility Treatment-Induced Stevens-Johnson Syndrome with a Severe Ocular Complication. *J Dermatol* (2019) 46(11):1042-5. Epub 2019/09/07. doi: 10.1111/1346-8138.15072.
18. Li L, Zheng S, Chen Y. Stevens-Johnson Syndrome and Acute Vanishing Bile Duct Syndrome after the Use of Amoxicillin and Naproxen in a Child. *J Int Med Res* (2019) 47(9):4537-43. Epub 2019/08/27. doi: 10.1177/0300060519868594.
19. Cao L. A Case Report of Plasma Exchange for Toxic Epidermal Necrolysis. *Anhui Medical and Pharmaceutical Journal* (2019) 23(07):1434-6+88.
20. Rao S, Li B, Li J, Xie H, Chen M, Zhao S, et al. Efficacy of Combined Lymphoplasma Exchange for the Treatment of Severe Refractory Immune-Related Skin Diseases: A Clinical Observation. *Chinese Journal of Dermatology* (2019) 52(1):16-9. doi: 10.3760/cma.j.issn.0412-4030.2019.01.004.
21. Han F, Zhang J, Hou YL, Wang XP, An JG, Wang XC, et al. Efficacy of Single-Session Plasmapheresis Therapy Alone for the Treatment of Toxic Epidermal Necrolysis in 17 Cases: A Clinical Observation. *Chinese Journal of Dermatology* (2018) 51(12):896-8. doi: 10.3760/cma.j.issn.0412-4030.2018.12.010.
22. Santosa, Rumbiana A, Wahab Z, Kurniawan SP, Naibaho RM, Yogyartono P. Successful Treatment of Methampyrone-Induced Toxic Epidermal Necrolysis with Therapeutic Plasma Exchange. *Case Rep Med* (2018) 2018:2182604. Epub 2018/08/21. doi: 10.1155/2018/2182604.
23. Jarrett B, Ghazala S, Chao J, Chaudhary S. Case of Steven-Johnson Syndrome in a Male with Breast Cancer Secondary to Docetaxel/Cyclophosphamide Therapy. *BMJ Case Rep* (2016) 2016. Epub 2016/11/18. doi: 10.1136/bcr-2016-217255.
24. Hung PC, Wang HS, Hsia SH, Wong AM. Plasmapheresis as Adjuvant Therapy in Stevens-Johnson Syndrome and Hepatic Encephalopathy. *Brain Dev* (2014) 36(4):356-8. Epub 2013/06/20. doi: 10.1016/j.braindev.2013.05.010.
25. Aihara Y, Oyama Y, Ichikawa K, Takeshita S, Takahashi Y, Kambara T, et al. Toxic Epidermal Necrolysis in a 4-Year-Old Boy Successfully Treated with Plasma Exchange in Combination with Methylprednisolone and I.V. Immunoglobulin. *J Dermatol* (2012) 39(11):951-2. Epub 2012/03/15. doi: 10.1111/j.1346-8138.2012.01534.x.

26. Koštál M, Bláha M, Lánská M, Koštálová M, Bláha V, Štepánová E, et al. Beneficial Effect of Plasma Exchange in the Treatment of Toxic Epidermal Necrolysis: A Series of Four Cases. *J Clin Apher* (2012) 27(4):215-20. Epub 2012/03/13. doi: 10.1002/jca.21213.
27. Szczeklik W, Nowak I, Seczynska B, Sega A, Krolikowski W, Musial J. Beneficial Therapeutic Effect of Plasmapheresis after Unsuccessful Treatment with Corticosteroids in Two Patients with Severe Toxic Epidermal Necrolysis. *Ther Apher Dial* (2010) 14(3):354-7. Epub 2010/07/09. doi: 10.1111/j.1744-9987.2009.00800.x.
28. Arvidson J, Kildal M, Linde T, Gedeborg R. Toxic Epidermal Necrolysis and Hemolytic Uremic Syndrome after Allogeneic Stem-Cell Transplantation. *Pediatr Transplant* (2007) 11(6):689-93. Epub 2007/08/01. doi: 10.1111/j.1399-3046.2007.00743.x.
29. Nomura T, Abe R, Fujimoto K, Endo T, Shimizu H, Koike T. Plasma Exchange; a Promising Treatment for Toxic Epidermal Necrolysis with Aids. *Aids* (2004) 18(18):2446-8. Epub 2004/12/29.
30. Yamada H, Takamori K, Yaguchi H, Ogawa H. A Study of the Efficacy of Plasmapheresis for the Treatment of Drug Induced Toxic Epidermal Necrolysis. *Ther Apher* (1998) 2(2):153-6. Epub 1999/05/04. doi: 10.1111/j.1744-9987.1998.tb00094.x.
